# Pulmonary Adenocarcinoma Arising From Somatic-type Malignant Transformation of a Nonseminomatous Germ Cell Tumor: A Case Report

**DOI:** 10.1177/23247096261488201

**Published:** 2026-09-03

**Authors:** Abdel Martinez, Brandon Cantazaro, Fatimah Bello, Christine Vega, Natacha Vargas, Payal Bhakta, Renzo Arauco

**Affiliations:** 1 441554 The University of Texas Rio Grande Valley School of Medicine, Weslaco TX, USA; 2 Internal Medicine, 20882 Knapp Medical Center, Weslaco TX, USA; 3 731797 Pulmonary & Sleep Center of the Valley, Weslaco TX, USA

**Keywords:** pulmonary adenocarcinoma, somatic-type malignant transformation of a nonseminomatous germ cell tumor

## Abstract

Somatic-type malignant transformation (STM) is a rare but aggressive complication of nonseminomatous germ cell tumors (NSGCTs), most commonly arising from the teratomatous component and frequently occurring in metastatic sites. We report the case of a man in his 20s with a history of mixed NSGCT (50% yolk sac tumor, 40% teratoma, and 10% embryonal carcinoma) treated with left orchiectomy and chemotherapy in 2023, who initially responded to treatment but was subsequently lost to follow-up. He presented with one week of progressive dyspnea and pleuritic left-sided chest pain. Serum alpha-fetoprotein was markedly elevated at 2,966.7 ng/mL. Computed tomography demonstrated a progressive left hilar mass causing near-complete compression of the left mainstem bronchus with postobstructive consolidation, pulmonary vein tumor thrombus extending into the left atrium, a stable subcarinal metastatic mass, and a small left pleural effusion. Brain magnetic resonance imaging and transthoracic echocardiography were unremarkable except for normal cardiac function without intracardiac thrombus. CT-guided biopsy of the lung mass demonstrated adenocarcinoma consistent with a pulmonary phenotype. Following multidisciplinary evaluation, the lesion was determined to represent pulmonary adenocarcinoma arising from somatic-type malignant transformation of the teratomatous component of recurrent NSGCT. The patient remained clinically stable and was managed with close airway surveillance after Pulmonary and Critical Care Medicine consultation, with airway stenting or endobronchial debulking reserved for clinical deterioration. This case highlights the importance of recognizing STM in patients with recurrent NSGCT, as accurate diagnosis requires integration of clinical history, imaging, and pathology and has significant therapeutic and prognostic implications.

## Background

Testicular germ cell tumors (TGCTs) are the most common solid malignancy affecting men between 15 and 40 years of age. Nonseminomatous germ cell tumors (NSGCTs) comprise a heterogeneous group of neoplasms that frequently contain multiple histologic components, including embryonal carcinoma, yolk sac tumor, choriocarcinoma, and teratoma. Although cisplatin-based chemotherapy has dramatically improved survival, recurrent or metastatic disease remains an important cause of morbidity and mortality, particularly in patients with chemotherapy-resistant tumors or late relapse.^1,2^

Somatic-type malignant transformation (STM), previously referred to as teratoma with malignant transformation, is a rare but well-recognized phenomenon in which a germ cell tumor differentiates into a histologically distinct non-germ cell malignancy. STM occurs in approximately 2%–6% of testicular germ cell tumors and most commonly arises from the teratomatous component, although it has also been described in association with yolk sac tumor. These transformations are encountered more frequently in metastatic lesions than in the primary testicular tumor and are associated with an aggressive clinical course and poorer prognosis.^2-5^

A broad spectrum of somatic malignancies has been reported, including sarcomas, carcinomas, primitive neuroectodermal tumors, nephroblastoma-like tumors, and hematologic malignancies. Among metastatic lesions, adenocarcinoma represents one of the most common epithelial subtypes of somatic-type malignant transformation. Because these tumors often exhibit morphologic and immunohistochemical features indistinguishable from primary carcinomas arising in the involved organ, correlation with the patient’s clinical history, prior germ cell tumor pathology, and molecular findings, including isochromosome 12p when available, is essential to establish the correct diagnosis.^2,3^

Unlike conventional germ cell tumors, somatic-type malignant transformations generally demonstrate limited responsiveness to platinum-based chemotherapy, making complete surgical resection the preferred treatment whenever feasible. Management frequently requires a multidisciplinary approach involving medical oncology, thoracic surgery, interventional pulmonology, pathology, and radiation oncology. Despite aggressive treatment, metastatic somatic-type malignant transformation remains associated with a poor prognosis, particularly when complete surgical resection cannot be achieved.^2-5^

Herein, we present a rare case of relapsed mixed NSGCT with pulmonary adenocarcinoma arising from somatic-type malignant transformation of the teratomatous component, presenting as a large hilar mass causing near-complete left mainstem bronchial compression, pulmonary vein tumor thrombus extending into the left atrium, and postobstructive pulmonary consolidation.

## Case

A man in his 20s with a history of a mixed nonseminomatous germ cell tumor (NSGCT), whose orchiectomy specimen demonstrated 50% yolk sac tumor, 40% teratoma, and 10% embryonal carcinoma, underwent left radical orchiectomy followed by chemotherapy after his initial diagnosis in 2023. His medical history was also significant for deep vein thrombosis and pulmonary embolism diagnosed in 2024. He had previously been treated with apixaban, which was discontinued because of significant hematuria, prompting placement of an inferior vena cava (IVC) filter. Although he initially demonstrated a favorable response to chemotherapy, he was subsequently lost to follow-up for more than one year.

He presented with one week of progressive shortness of breath and left-sided pleuritic chest pain that had worsened over the preceding 12 hours. On presentation, he was hemodynamically stable and maintained adequate oxygenation on room air. Laboratory studies revealed a white blood cell count of 6.64 × 10^3^/µL, hemoglobin of 13.5 g/dL, platelet count of 371 × 10^3^/µL, sodium of 141 mmol/L, potassium of 4.3 mmol/L, creatinine of 1.16 mg/dL, procalcitonin of 0.05 ng/mL, and alpha-fetoprotein (AFP) of 2,966.7 ng/mL.

**Figure 1.**
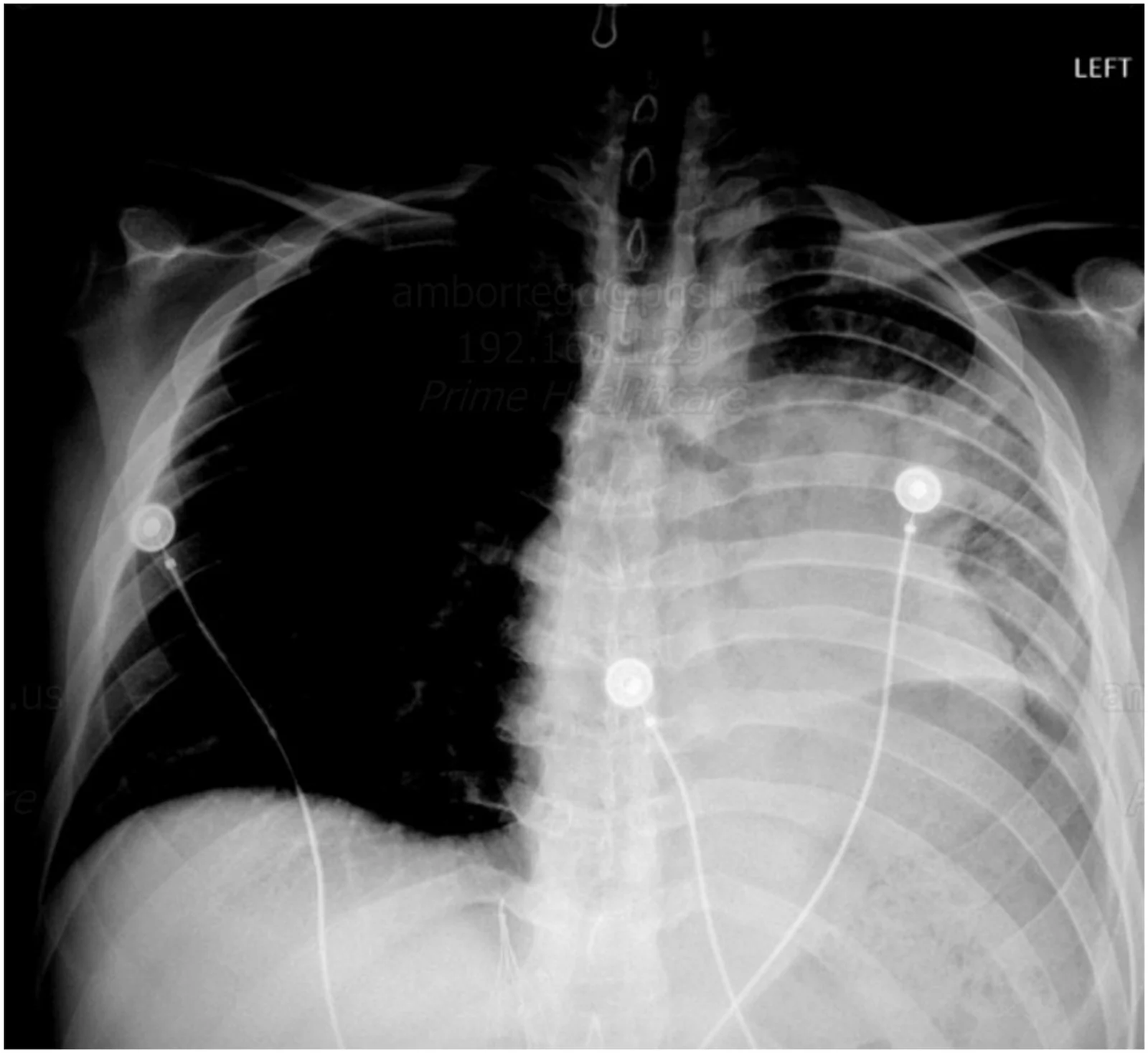
Chest radiograph demonstrating asymmetric left perihilar and lower lobe airspace opacities concerning for an infectious or neoplastic process

**Figure 2.**
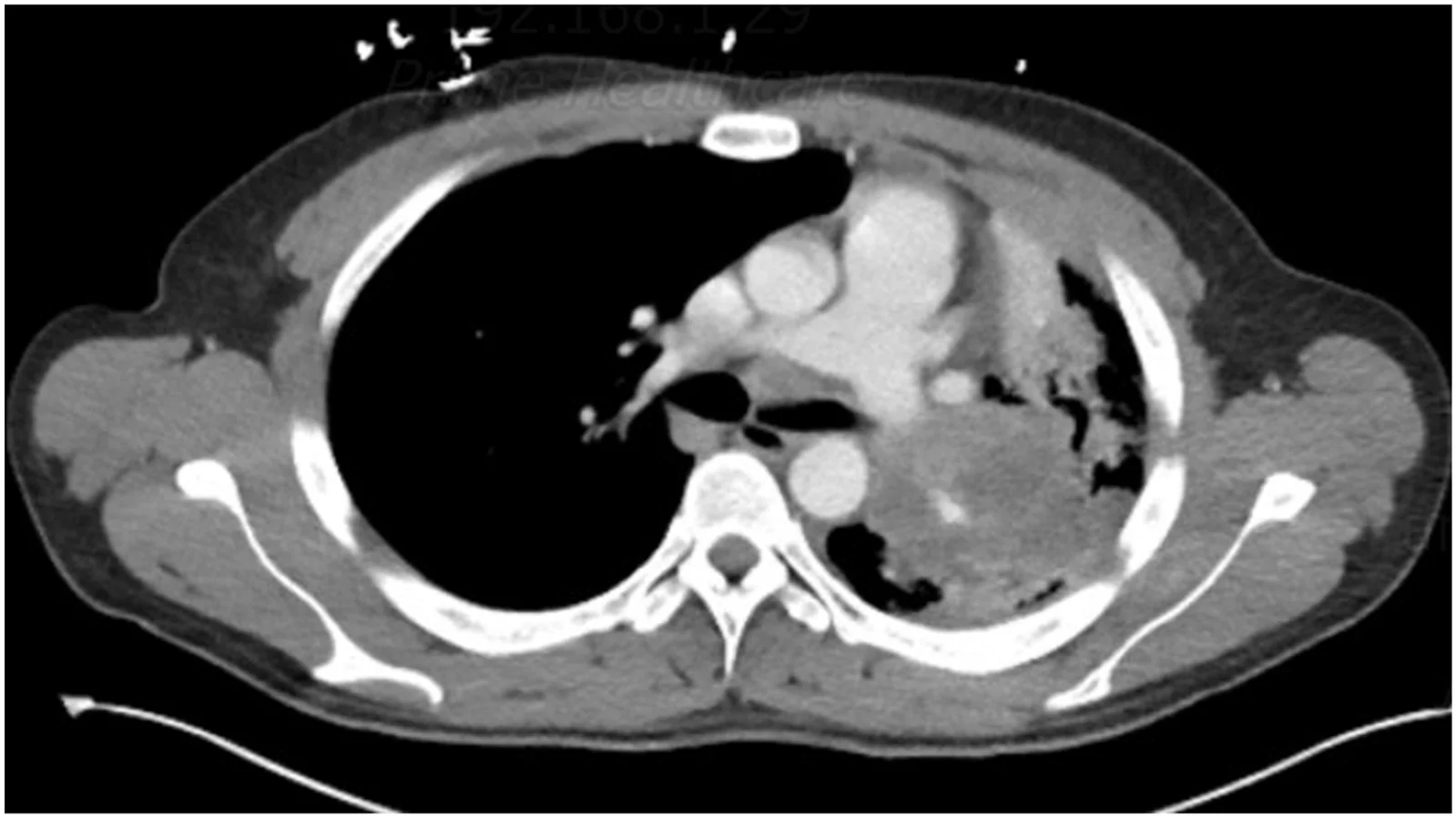
Contrast-enhanced computed tomography (CT) of the chest demonstrating a progressive left hilar mass measuring 6.8 × 5.5 cm

Chest radiography demonstrated asymmetric left perihilar and lower lung airspace opacities concerning for either an infectious or neoplastic process (Figure 1). Contrast-enhanced computed tomography (CT) of the chest, abdomen, and pelvis revealed a progressive left hilar mass (Figure 2) encasing the left upper and lower lobe bronchi with worsening postobstructive consolidation involving the left upper and lower lobes. Filling defects were identified within the left superior and inferior pulmonary veins, consistent with tumor thrombus extending into the left atrium. A stable subcarinal mass was compatible with metastatic disease. Additional findings included a progressive small left pleural effusion and hepatomegaly without evidence of acute intra-abdominal or intrapelvic abnormalities.

Transthoracic echocardiography demonstrated normal left ventricular systolic function with an ejection fraction of 60%, normal right ventricular size and systolic function, and no evidence of left ventricular thrombus. Magnetic resonance imaging (MRI) of the brain showed no acute intracranial abnormalities.

The patient subsequently underwent CT-guided biopsy of the left lung mass. Histopathologic examination of the left pleural mass biopsy demonstrated adenocarcinoma with a pulmonary phenotype. Immunohistochemical staining showed tumor cells positive for TTF-1 and negative for CK7, CK20, synaptophysin, and chromogranin, supporting the diagnosis of pulmonary adenocarcinoma. Following multidisciplinary review, the Oncology service concluded that these findings were most consistent with relapsed nonseminomatous germ cell tumor with pulmonary adenocarcinoma arising from somatic-type malignant transformation of the teratomatous component.

This conclusion was based on the integration of the patient’s clinical history, serologic findings, and pathology. The patient had a prior diagnosis of nonseminomatous germ cell tumor with markedly elevated AFP levels (922.3 ng/mL in 2024), persistent elevation during outpatient oncology follow-up, and a substantial increase to 2,966.7 ng/mL during the current admission, supporting relapse of the germ cell tumor. Furthermore, the patient’s young age (in his 20s) and absence of a cigarette smoking history, with only occasional prior marijuana use, made a primary pulmonary adenocarcinoma less likely. Collectively, the histopathologic findings and clinical context supported the diagnosis of pulmonary adenocarcinoma arising through somatic-type malignant transformation of the teratomatous component of a relapsed nonseminomatous germ cell tumor. The patient had previously received paclitaxel, ifosfamide, and cisplatin (TIP) chemotherapy, with an initial favorable response before being lost to follow-up.

Pulmonary and Critical Care Medicine was consulted for evaluation of the postobstructive pulmonary process and pleural effusion. The patient had recently completed a course of antibiotics for pneumonia prior to admission, and given the absence of clinical, laboratory, or radiographic evidence of an active infection, he was monitored off antibiotics during this hospitalization. Bedside point-of-care ultrasonography (POCUS) demonstrated only a minimal left pleural effusion and a large heterogeneous mass posterior to the heart. Imaging also demonstrated near-complete collapse of the left mainstem bronchus secondary to extrinsic compression from the hilar mass. Because the patient remained hemodynamically stable and without significant respiratory compromise, a multidisciplinary decision was made to closely monitor his airway. Interventional bronchoscopic therapies, including airway stent placement and endobronchial tumor debulking, were considered should he develop worsening airway obstruction or respiratory compromise. The patient was managed conservatively for five days, after which repeat chest imaging was obtained to reassess the airway obstruction and evaluate for interval progression of the lesion.

## Conclusion

Somatic-type malignant transformation of a nonseminomatous germ cell tumor is an uncommon but clinically significant mechanism of disease progression that should be considered in patients with a history of NSGCT who develop enlarging or recurrent masses despite prior chemotherapy. This case highlights the importance of maintaining a broad differential diagnosis when new pulmonary lesions are identified in survivors of testicular cancer, particularly in the setting of teratomatous elements within the primary tumor. Accurate diagnosis requires integration of the patient’s oncologic history, imaging findings, histopathology, and multidisciplinary evaluation to distinguish somatic-type malignant transformation from a second primary malignancy. Early recognition is essential, as these tumors often exhibit resistance to conventional germ cell tumor chemotherapy and may require individualized management, including consideration of surgical resection or histology-directed therapy. This case also illustrates the potential for life-threatening local complications, including central airway compression and pulmonary vein tumor thrombus with left atrial extension, underscoring the need for coordinated multidisciplinary care involving oncology, pulmonary and critical care medicine, interventional pulmonology, radiology, pathology, and thoracic surgery.

## References

[1] Gilligan T Lin DW Adra N , et al. NCCN Guidelines® Insights: Testicular Cancer, Version 2.2025. J Natl Compr Canc Netw. 2025;23(4):e250018. 40203876 10.6004/jnccn.2025.0018

[2] Guo CC Czerniak B . Somatic-type malignancies in testicular germ cell tumors. Hum Pathol. 2022;127:123-135. 35803413 10.1016/j.humpath.2022.06.024

[3] Hwang MJ Hamza A Zhang M , et al. Somatic-type malignancies in testicular germ cell tumors: a clinicopathologic study of 63 cases. Am J Surg Pathol. 2022;46(1):11-17. 34334690 10.1097/PAS.0000000000001789 PMC8671201

[4] Motzer RJ Amsterdam A Prieto V , et al. Teratoma with malignant transformation: diverse malignant histologies arising in men with germ cell tumors. J Urol. 1998;159(1):133-138. 9400455 10.1016/s0022-5347(01)64035-7

[5] Cheema A Siddiqui F Kamran A . Germ cell tumor with somatic-type malignancy: a case report and review of the literature. Cureus. 2022;14(6):e25879. 35844345 10.7759/cureus.25879 PMC9278484

